# The level of formal support received by people with severe mental illness living in supported accommodation and participation: A systematic review

**DOI:** 10.1177/0020764020988576

**Published:** 2021-01-25

**Authors:** Akkara Lionel Jose, Michele Harrison, Anusua Singh Roy, Linda Irvine- Fitzpatrick, Kirsty Forsyth

**Affiliations:** 1 School of Health Sciences, Queen Margaret University, Queen Margaret University Drive, Edinburgh, UK; 2 School of Health Sciences, Queen Margaret University, Edinburgh, UK; 3 Strategic Programme Manager, Mental Health and Wellbeing, City of Edinburgh Health and Social Care Partnership, Edinburgh, UK

**Keywords:** Supported accommodation, social participation, empowerment, daily living functioning, severe mental illness

## Abstract

**Aim:**

The review aimed to identify and explore the association of level of support received by people with severe mental illness in supported accommodation and participation.

**Method:**

The authors conducted a systematic search in MEDLINE, PsychINFO, PsychARTICLES, CINAHL Plus and ASSIA. Searches were restricted to articles published in English and participants aged 18 years and over with severe mental illness. Articles were included based on level of support received in mental health supported accommodation, classified according to the Simple Taxonomy for Supported Accommodation, and three factors of participation: social participation, daily living functioning and personal empowerment. Studies of in-patient settings and nursing homes were excluded. The review protocol is registered on PROSPERO (registration number: CRD42019161808).

**Results:**

Six articles were included in the review from USA, Australia, Sweden and Taiwan. Factors of participation for people living in accommodation with moderate support and accommodation with high support were explored. Data indicated an association between level of support and participation showing that people living in accommodation with moderate support had increased participation compared to people living in accommodation with high support.

**Conclusion:**

This review identified an association between level of formal support and participation. People with SMI living in accommodation with medium support participated in more community occupations, more activities and had a higher level of personal empowerment than people living in accommodation with high support.

## Introduction

People with severe mental illness (SMI) have diagnoses such as schizophrenia, personality disorders, bipolar disorder and other psychosis-related disorders and have a range of complex needs which impact on different aspects of their everyday life. Supported accommodation (SA) provides residential, community-based support for individuals with SMI (McPherson et al., 2018a). SA provides individuals with SMI the opportunity to obtain a tenancy while receiving varying levels of staff support within the least restrictive settings in order to develop skills and abilities needed to participate in various daily living and social activities (Padmakar et al., 2020). SA can differ by type, staffing location, level of support provided and emphasis within the accommodation on moving on (McPherson et al., 2018b). Within SA, the support people with SMI receive is typically provided by formal carers inclusive of healthcare professionals, carers or other staff providing support.

Participation can vary over a person’s lifetime dependant on life events affecting the person’s confidence, abilities and motivation (Sánchez et al., 2016). For people with SMI, this can affect maintaining and creating relationships with friends and family (Cruce et al., 2012), how they engage with the support they receive and being in education or work and pursuing interests (Tjörnstrand et al., 2013). Participation therefore has several elements. These include engaging in daily living activities (self-care, meal planning and preparation, dressing, money management, medication management (Piškur et al., 2014); social participation, an individuals’ involvement in society (Sanches et al., 2019) through roles they engage in within a group or in their community (Kaplan et al., 2012), including employment and vocational activities (van Eijk-Hustings et al., 2013), social functioning (Tobin et al., 2013) and building and maintaining relationships (Berkman, 2001); and personal empowerment, the feeling or sense of control an individual has over their own life alongside the level of responsibility and autonomy they possess to initiate and act on aspects of their participation (Bruschetta & Barone, 2016; Cavalieri & Almeida, 2018).

Literature focused on SA suggest when accommodation types or treatment environments are appropriate to people’s needs there are improvements in activities of daily living and social participation (Siskind et al., 2012). It has also been shown that the therapeutic relationship between people living in SA and formal carers can improve social participation (Amati et al., 2017; Brunt & Rask, 2018; Krotofil et al., 2018). This results in increased personal and social responsibility for the individual and improved social functioning (Dixon et al., 2016; Green et al., 2009; Hitch et al., 2013). Previous systematic reviews have focused on the effect of the built and physical environment on mental health (Charlotte et al., 2007; Moore et al., 2018) and the impact of social climate, service delivery and quality of life for people with SMI living in SA. These factors have been shown to affect how care provided meets the person’s needs (Macpherson et al., 2004), people with SMI’s experience and satisfaction with SA (Harrison et al., 2020; Krotofil et al., 2018) and the impact on individual’s feelings of stability and independence (Burgoyne, 2014). Reviews also focused on factors such as SA’s links to psychosocial outcomes (McPherson et al., 2018a), quality or effectiveness of service delivery style (Rogers et al., 2010) and standardising service delivery models (Parker et al., 2019; Tabol et al., 2010). There is, however, no systematic review that considers formal support for people with SMI living in SA and its association with factors of participation.

The systematic review aimed to review formal care provided to people with SMI living in SA and its association with factors of participation specifically daily living functioning, social participation and personal empowerment.

## Methods

The following review protocol is registered on PROSPERO (registration number is CRD42019161808) and follows the PRISMA guidance.

### Eligibility criteria

The review included articles published in academic journals with quantitative data relevant to the three participation factors: daily living functioning, social functioning and personal empowerment. Inclusion criteria were adults with SMI living in SA, receiving support from formal carers ( nurses, paid carers and/or any health care professionals) and informal carers (family, friends or unpaid carers) Dissertations, book chapters, guidelines, policy and conference proceedings were excluded from the study. Studies reporting on people under 18 years old and those that were not published in English were excluded, however, no exclusions were made based on country of publication. Studies within in-patient settings, nursing homes and SA that was not being provided to people with SMI were excluded from the review.

### Population

This review includes people with SMI aged 18 and above. The term SMI extends to the DSM-IV definition and includes the following conditions: schizophrenia, bipolar disorder, personality disorder or other psychosis-related disorders. Diagnoses were reviewed during the screening process to comply with inclusion criteria. Studies were excluded if they reported solely on the following diagnoses: substance misuse, eating disorders, learning/intellectual disability or dementia.

### Supported accommodation

SA was classified using the Simple Taxonomy for Supported Accommodation (STAX-SA) (McPherson et al., 2018b) which defines accommodation types by staffing location (on or off site), level of support (high/moderate/low/no), emphasis on move-on (limited or strong) and physical setting (congregate or individuals).

### Formal support

Formal support was defined using the STAX-SA level of support domain. The four levels of support (high/moderate/low/none) describe the frequency, nature and intensity of support (including stafﬁng duration) required to meet service user need (e.g. for personal care, medication management). Studies identified with moderate support where available staff where identified as on or off site were combined for the purpose of the review.

### Comparator

The search strategy reflects the authors’ initial aim to explore formal care compared with informal care. Due to the limited information and lack of consistency of informal support provision detailed within studies, this comparator was not used. Studies were instead compared according to level of support and their association with participation for people with SMI living in SA.

### Outcomes

Three participation factors, social participation, daily living functioning and personal empowerment were identified, with reported data in the included studies matched to these three factors (see Table 1). Detailed information about how factors were matched and measures used in included articles is available in Supplemental Material 1.

**Table 1.** Data extraction table for included studies.

Author, year, country	Study design	Level of support	Sample size	Mean age (years)	Diagnoses	Gender	Factors of participation			Study quality: measure/rating
							Social participation	Daily living functioning	Personal empowerment	
Kruzich and Berg (1985), America	Cohort study	High	42	Mean not reported. Over half were 35–65; 25% over 66 and a fourth under 35*	66% schizophrenia, 7% affective disorder, 16% Chronic brain disorder, 10% with other mental health conditions	Male 51% Female 49%	–	Self sufficiency	–	RTI: risk of bias unclear.
		Moderate	21							
		None	20							
Nelson et al. (1997), Canada	Quasi-experimentalnon-equivalent comparison group design.	High	30	32.5	Inclusion criteria: Psychiatric consumers/ survivors.	Male 60% Female 40%	Instrumental roles	Independent functioning	Mastery	ROBINS-I: moderate risk of bias
		Moderate	52	34.1		Male 67% Female 33%				
		None	25	45		Male 44% Female 56%				
Fossey et al., (2006), Australia	Cross-sectional study	High	25	35.09	Schizophrenia	Male 56% Female 44%	Social contact	Self-care	Responsibility	RTI: low risk of bias
		Moderate	18							
Dorer et al. (2009), UK	Cross-sectional study (staff-survey method)	High	91	42.7	Unspecified Long-term mental health problems	Male 66% Female 34%	Education, employment, day centre, local facilities, faith, family and friends	–	–	RTI: high risk of bias
		Moderate	62							
Shu et al. (2001), Taiwan	Quasi-experimental (Longitudinal design)	High	23	37.8	Schizophrenia	Male 57% Female 43%	Social activity	–	Autonomy	ROBINS-I: moderate risk of bias
		Moderate	37	32.4						
Eklund and Tjörnstrand (2019), Sweden	Cross sectional (secondary data analysis)	High	155	48	62% psychosis, 13% anxiety/mood disorder and 25% other mental health disorder	Male 46% Female 64%	Employment status, education status, work training, day centre, leisure activities, cultural activities	Doing household work, managing self-care, gardening and repairs, Physical exercise.	–	RTI: low risk of bias
		Moderate	111	46						

*Note*. RTI **=** Research Triangle Institute item bank; ROBINS-I **=** Risk Of Bias In Non-randomised Studies of Interventions.

* As reported by study.

### Search strategy

An electronic database search was conducted between October 2019 and February 2020 using MEDLINE, Psychinfo, CINAHL Plus, ASSIA and PsychARTICLES. Alongside this, previous reviews were hand-searched to identify any relevant articles. The searches included a combination of MeSH terms and Boolean phrases that matched the population, interventions, comparators and outcomes. These included, but were not limited to, ‘Mental health difficult*’, ‘Shared accommodation’, ‘social interaction/engage*’, ‘formal care provision’, ‘Formal Support’, ‘informal support’, ‘Factors of participation’, ‘improved skills and abilities’ and ‘engage*’. No time limit was placed on publication date however articles were limited to population ages of 18 and above. The full search strategy used within the databases is detailed in Supplemental Material 2.

### Data extraction

Data was extracted according to a form developed by ALJ, and included (1) Study title, year, location, study type; (2) Sample size, age, gender, condition/inclusion criteria; (3) Accommodation type, support type (formal or informal); (4) Factors of participation, measures used, control/comparators; (5) Statistical analysis and findings/results. The extracted data was synthesised to include identified inclusion criteria and are detailed in Table 1. Statistical information pertaining to accommodation types with relevant level of support was included and is detailed in Table 2.

**Table 2.** Data extraction of results from selected studies.

Study	Level of support	Sample size	Factors and data reported										
			Social participation						Daily living functioning				Personal empowerment
Kruzich and Berg, (1985), America									Self-sufficiency (mean)				
	High	42	–						1.23				**–**
	Moderate	21							2.19					None	24							3.04			
	Nelson et al. (1997), Canada			Instrumental Roles (mean)						Independent functioning (mean)			
Mastery (mean)		High	30	0.73						14.3			
20.4		Moderate	52	0.89						16.1			
21.3		None	25	0.56						11				18.6
Fossey et al. (2006), Australia	High	25	Social Contact (mean)						Self-care (mean)			
			Responsibility (mean)	15.8						31.88			
	16.96	Moderate	18	17						32				17.76
Dorer et al. (2009), UK			Estimated time for participation in specific community occupations (Mean in Hours (SD))									
				Education	Employment	Day centre	Local facilities	Faith	Family and friends	–			
	–	High	91	0.6 (2.6)	0.4 (1.5)	0.8 (3.2)	6.2 (7.7)	0.3 (1.2)	8.1 (16.9)				
		Moderate	62	1.7 (4.2)	1.0 (4.1)	1.9 (3.9)	8.0 (9.1)	0.1 (0.6)	13.9 (24.3)					
Shu et al. (2001), Taiwan			Social activity factor (GEE regression coefficient)*									
	Autonomy Factor (GEE Regression Coefficient)**	High	23	1.62, *p* = .01						–			
	1.20, *p* = .04	Moderate	37											
Eklund and Tjörnstrand (2019), Sweden			Percentage of participants involved in occupations						Percentage of participants involved in occupations			
		High	155	Employed or a student	Training or enrolled in studies	Attending a day centre	Organised leisure/hobbies	Work training	Cultural occupations	Doing household work	Gardening or repairs	Managing own personal hygiene daily	Physical exercise
	**–**	Moderate	111	9%	10%	41%	23%	11%	79%	83%	10%	87%
				88%	11%	14%	37%	34%	15%	79%	72%	37%	87%	88%

*Note.* GEE = generalised estimating equations; SD = standard deviation.

* Social activity between group for level of support.

### Quality assessment

The quality of the four observational studies was assessed using the Research Triangle Institute (RTI) Item Bank (Viswanathan & Berkman, 2011; Viswanathan et al., 2013) due its ability to comprehensively assess bias (selection, performance, detection and confounding) across varying types of observational studies. A total of 11 questions were selected as appropriate to assess the risk of bias for the included studies. Studies with one or more negative score were recorded as having a high risk of bias and those which scored 1 or more ‘partially’ or ‘cannot determine’ were recorded as having an unclear risk of bias (Viswanathan & Berkman, 2011). The ROBINS-I tool was used for the two quasi-experimental studies selected (Sterne et al., 2016). The tool is an update to the Cochrane collaboration risk of bias tool assessing seven domains of bias at the pre-intervention, at-intervention and post-intervention stages of a study (Sterne et al., 2016). Risk of bias of individual studies was assessed independently by ST and ALJ. Overall scores are presented in Table 1 and detailed results are available in Supplemental Material 3.

### Data synthesis

Data collected could not be synthesised within a meta-analysis due to inconsistency of data reporting, unavailability of data required to calculate a common effect size (Cohen’s d; Cohen, 1988) and use of unstandardised measures of participation with no evidence of reliability or validity testing. Contacting authors for additional information or data was unsuccessful due to no response or the author no longer possessing the original data. Alternative methods to a meta-analysis recommended in the Cochrane guidelines (Deeks et al., 2019, Higgins et al., 2019) were used to include statistical data to support the systematic review, by calculating actual or estimated effect sizes where possible. Data synthesis was completed by ALJ. As seen in Table 3, data available was used to calculate Cohen’s d (Cohen, 1988; Dorer et al., 2009; Fossey et al., 2006) and a raw mean difference (Nelson et al., 1997) alongside the ANOVA value (Nelson et al., 1997) and a regression coefficient (Shu et al., 2001). These were used to estimate the magnitude, direction and statistical significance of association between level of support and level of participation as well as the association of the participation factors within specific levels of support. Cohen’s d was calculated for one study (Fossey et al., 2006) using the ‘dmetar’ package (Harrer et al., 2019) in R (R Core Team, 2013), as t-tests were calculated for sub-group difference in this study. Reporting of data uses the SWIM guidelines (Campbell et al., 2020) which provides additional structure for reporting of the narrative synthesis of the systematic review while adhering to the PRISMA checklist.

**Table 3.** Calculated effect sizes.

Factor	Level of support	No. of studies	Sample size (total)	Results	
Social participation	High versus moderate	5	604		
Instrumental roles				RMD = –0.16	
Social contact				*d* = –0.3957	
				Sub-group difference (*t*) = –1.28 *p* = .21	
				CI = –3.10 to 0.70	
Estimated time for participation in specific community occupations				Education: *d* = −0.321	Combined SMD* (*d*): –0.1926 *p* = .004
				Employment: *d* = −0.210	
				Day centre: *d* = –0.31	Fixed effects model *p* < .05, CI = –0.3250 to –0.0603
				Local facilities: *d* = –0.21	
				Faith: *d* = 0.199	
				Family and friends: *d* = –0.28	
Social activity factor				GEE regression coefficient between group: 1.20, *p* = .04	
Percentage of participants involved in daily activities				Employed or student(PD) = –2%,	
				Working or enrolled (PD) = –4%	
				Day centre (PD) = 4%	
				Leisure/hobbies (PD) = –11%,	
				Work training (PD) = –4%	
				Cultural occupation (PD) = 0%	
	High and moderate versus no	1	25		
Instrumental roles				*F*(2,104) = 3.0, *p* < .05**	
Daily living function	High versus moderate		454		
Self-sufficiency				RMD = –0.96	
Independent functioning				RMD = –1.8	
Self-care				*d* = –0.022	
				Sub-group difference (*t*) = –0.07	
				*p* = .9, CI=–3.49 to 3.25	
Percentage of participants involved in daily living activities				Household work (PD) = 11%	
				Gardening (PD) = –27%	
				Personal hygiene (PD) = 0%	
				Physical exercise (PD) = 0%	
	High and moderate versus no	2	194		
Self-sufficiency				RMD = 0.38	
Independent functioning				*F*(2,101) = 6.4, *p* < .001	
Personal empowerment	High versus moderate	3	185		
Mastery				RMD = –0.9	
Responsibility				*d* = –0.329. Sub-group difference (*t*) = –1.04 (*p* = .30); CI: –2.37 to 0.77	
Autonomy				GEE reg. coefficient between group = 1.62, *p* = .01	
	High and moderate versus no	1	107		
Mastery				*F*(2,96) = 3.1, *p* < .05**	

*Note. d* = Cohen’s d; RMD = raw mean difference; PD = percentage difference.

* SMD = standardised mean difference.

** ANOVA for group.

## Results

### Study selection

**Figure 1.**
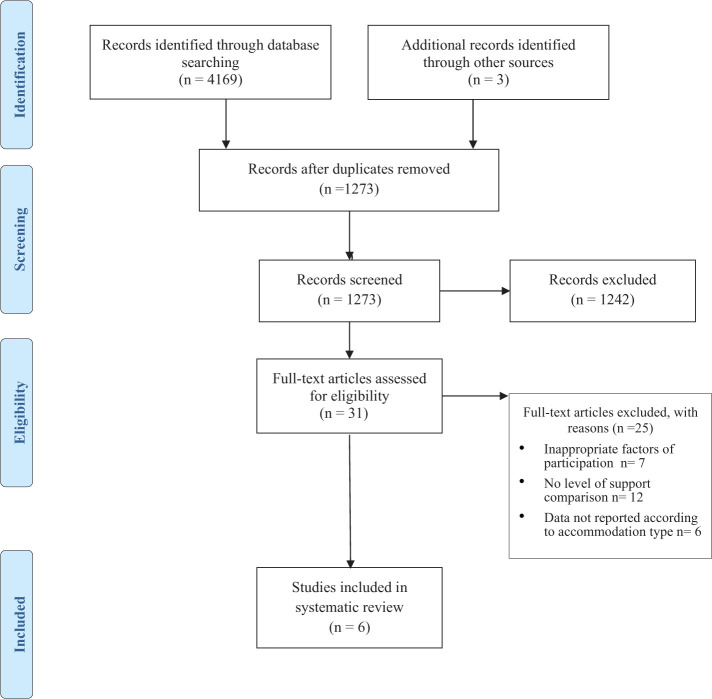
PRISMA flow diagram.

An initial search using the search strategy above presented 7,892 articles from Medline, PsychINFO, PsychARTICLES, CINAHL using EBSCOHOST and 222 from ASSIA. After adding filters for age and language the results were 3,948 (EBSCOHOST) and 221 (ASSIA). Duplicates were then removed from the initial search resulting in 1,270 results. Following this titles and abstracts were screened and the results of this were reviewed for relevance by ST. ST reviewed 10% against the inclusion/exclusion criteria. Discrepancies (less than 5%) between ST’s and ALJ’s results were discussed and resolved. The screening process can be seen in Figure 1.

Following title, abstract and full-text screening the review identified six articles that addressed association between participation and at least two levels of support received by individuals with SMI. The review identified four observational studies using cross-sectional data and two quasi-experimental studies using longitudinal data. One of the observational studies was secondary analysis of cross-sectional data (Eklund and Tjornstrand 2019). Studies were conducted in America (*n* = 1), UK (*n* = 1), Australia (*n* = 1), Sweden (*n* = 1), Taiwan (*n* = 1) and Canada (*n* = 1; see Table 1).

### Quality appraisal

Heterogeneity was identified across the observational studies due to the difference in participants across the studies, with two studies reporting data from people with SMI only (Eklund & Tjörnstrand, 2019; Fossey et al., 2006); one study reporting data from staff only (Dorer et al., 2009) and one study reporting data from both people with SMI and staff (Kruzich & Berg, 1985). Two studies were analyses of secondary data (Eklund & Tjörnstrand, 2019) (Kruzich & Berg, 1985). None of the included studies shared a common measurement tool. Length of stay in accommodation was only reported in 2 studies (Eklund & Tjörnstrand, 2019; Nelson et al., 1997) so its potential effect on participation could not be considered.

Two studies (Eklund & Tjörnstrand, 2019; Fossey et al., 2006) were rated at low risk of bias; Kruzich and Berg’s (1985) study was rated ‘Unclear’ due to the recruitment strategy and attrition rate not being reported. Dorer et al.’s study (2009) was rated at high risk of bias as they did not use a validated measure. The studies were not excluded as Kruzich and Berg (1985) reported an internal consistency reliability coefficient and the measures used in Dorer et al.’s (2009) study were informed by existing standardised measures to improve content validity. Both quasi-experimental studies were found to have a moderate risk of bias. Nelson et al.’s (1997) study scored moderate on confounding bias due to measures for recording confounding variables being subjective, suggesting a higher risk of confounding bias. Shu et al.’s (2001) study scored a moderate in relation to reporting bias, as specific outcome data presented was not clearly labelled, so interpretation of values is assumed rather than stated.

### Participation factors

#### Social participation

Social participation was the most frequently identified factor present within five out of six studies. Five studies presented data comparing accommodation with high support (AHS) and accommodation with moderate support (AMS) and their association with social participation. Social participation described in these studies included people’s involvement in roles related to education and employment, frequency of participation in social activities during the week and time spent in social activities. The social participation factors reported show overall that people with SMI living in AMS participated in more community activities than those living in AHS, identifying more social roles (Nelson et al., 1997) and social contacts (Fossey et al., 2006) including visiting family and friends more frequently (Dorer et al., 2009). Actual and calculated effect sizes estimated demonstrated that people’s social participation was statistically significant in relation to higher level of engagement in social activities (Shu et al., 2001). The combined effect size of time spent in community occupations in Dorer et al.’s (2009) study was statistically significant, showing a small effect size and people with SMI in AMS identified more social roles (Nelson et al., 1997) and had more social contacts (Fossey et al., 2006). Two elements were the exception to this inference; ‘faith’ in Dorer et al’s (2009) study and ‘day centre’ in Eklund and Tjörnstrand’s (2019) study with individuals in AHS spending more time participating in these social activities than those in AMS.

Only one study (Nelson et al., 1997) compared social participation between a combined value of AHS and AMS with accommodation with no support (ANS). A mixed two-way ANOVA produced a statistically significant difference (*p* < .05) that suggests those in AHS and AMS identify more social roles than those in ANS.

#### Daily living functioning

Daily living function was identified in four of the studies (Eklund & Tjörnstrand, 2019; Fossey et al., 2006; Kruzich & Berg, 1985; Nelson et al., 1997) and compared between AMS and AHS. The direction of inference across all but one study (Eklund & Tjörnstrand, 2019) suggests that people with SMI living in AMS participate in more daily living activities than those in AHS. While no statistically significant association was identified between support type and elements of daily living functioning, values calculated and reported support this direction of inference. Eklund and Tjörnstrand’s (2019) study differs by reporting that 11% more individuals in AHS engage in household chores compared to those in AMS. Two studies reported data comparing a combined value of AHS and AMS with ANS. Kruzich and Berg (1985) calculated a raw mean difference of (0.38) for self-sufficiency, and Nelson et al. (1997) reported values from a two-way mixed ANOVA showing a statistically significant difference in daily living function, with people living in AHS and AMS being more independent in daily living functioning than those living in ANS.

#### Personal empowerment

Three studies reported data on personal empowerment in AHS and AMS (Fossey et al., 2006; Nelson et al., 1997; Shu et al., 2001). Personal empowerment identified in these studies included mastery, autonomy and responsibility. Calculated and available data suggests the direction of inference shows that people with SMI living in AMS have higher levels of personal empowerment than those in AHS. Shu et al.’s study (2001) reported an unstandardised regression coefficient for autonomy favouring those in AMS. In Fossey et al.’s (2006) study, effect size d calculated for ‘responsibility’ showed a small effect size, with no statistical significance for subgroup difference. Nelson et al. (1997) compared mastery, using a combined value for AHS and AMS with ANS. A two-way mixed ANOVA identified a statistical significance which showed that those in AMS and AHS had a higher level of personal empowerment than those in ANS.

## Discussion

The review identified an association between participation and level of support, particularly when comparing AHS and AMS. Social participation was the most frequently reported, followed by daily living functioning and personal empowerment. All factors demonstrated favourable results for people with SMI living in AMS who had higher levels of participation than those living in AHS, with few identified discrepancies in this direction of inference. Only two studies (Kruzich & Berg, 1985; Nelson et al., 1997) reported on ANS and compared this with a combined value for AHS and AMS, suggesting higher levels of social participation and daily living functioning in accommodation with support when compared to ANS.

The review identified that people with SMI living in AMS had higher levels of social participation. The studies included in this review suggest that those in AMS received more staff support to socially participate, particularly to attend community centres (Dorer et al., 2009; Eklund & Tjörnstrand, 2019) and enrol in vocational activities (Nelson et al., 1997) than those in AHS. Previous research suggests this may be due to range of factors including how services are structured, particularly facility size, whether staff are based on or off site, intensity of support provided and whether there is a focus on moving on to more independent living (Dalton-Locke et al., 2018; Hansson et al., 2002; Macpherson et al., 2012; Muir et al., 2010, Webber & Fendt-Newlin, 2017). It is reported that people with SMI living in AMS have higher levels of choice and freedom when compared to people living in AHS (Eklund & Tjörnstrand, 2019; Nelson et al., 1997). There is also discussion in the literature about whether higher levels of participation for people with SMI living in AMS are due to people having less complex needs including experiencing fewer symptoms and being on less medication (Segal et al., 1989; Shu et al., 2001, Killaspy et al., 2019), resulting in greater motivation to participate in activities (Nelson et al., 1997). However other studies have shown that level of disability in supported accommodation is comparable regardless of level of support received (Trauer, 2001; Trauer et al., 1997). Across the studies reviewed, the level of participation in employment or some form of education is low. This is reported in other studies of people with SMI living in supported accommodation (Bitter et al., 2016; Killaspy et al., 2016; Mirza et al., 2008). It is recognised that employment and education are important for social functioning for people with SMI (Modini et al., 2016) however the indication from this review is that this remains an area of social participation that is not available to many people with SMI when living in supported accommodation.

The review suggests that people with SMI living in AMS had higher levels of participation in daily living activities than those living in AHS. The minimal difference in mean scores and correlation statistics reviewed for daily living functioning factors between AMS and AHS is interesting as there is an increased focus in AMS on rehabilitation and increasing independence in daily living skills (Brunt & Hansson, 2002; Killaspy et al., 2016; Krotofil et al., 2018). All the studies described staff support in AHS as providing more guidance and support around daily living activities to people with SMI, with them receiving staff assistance with activities or high levels of prompting (Fossey et al., 2006; Kruzich & Berg, 1985; Nelson et al., 1997). Eklund and Tjörstrand’s (2009) study reported that people living in AHS participated in more household chores than those living in AMS. This may be due to people living in AHS spending more time in the accommodation as they had lower levels of social participation than people living in AMS, resulting in daily living activities being the main focus of daily time use. When results for daily living functioning in AHS and AMS were combined and compared to ANS, a positive association was demonstrated between accommodation with support and participation in daily living activities. It is generally assumed that people living in accommodation with no support are independently participating in all daily living activities (Trauer, 2001). However, research has shown that people with SMI receiving no or low support can experience difficulties organising daily living activities (Eklund et al., 2017).

Personal empowerment is the least explored factor among the selected studies. Results indicate a higher level of personal empowerment reported by people living in AMS compared to those living in AHS. Personal empowerment is an important aspect of recovery for people with SMI (Leamy et al., 2011). This review suggests people with SMI’s experience of personal empowerment will vary depending on the level of support they receive in supported accommodation, with studies reporting increased personal empowerment as the level of support decreases. This may be indicative of peoples’ perceptions of their own abilities and growth in confidence over time with people living in AMS in Nelson et al. (1997) and Shu et al.’s (2001) studies experiencing higher levels of personal empowerment as a result of increased independence and recovery. However, Nelson et al.’s (1997) study showed that personal empowerment related to skill mastery was lower in ANS compared to AMS and AHS. Research has shown that staff attitudes towards recovery influence people with SMI’s level of participation, inhibiting their recovery (Bitter et al., 2017; Linhorst et al., 2005; Macpherson et al., 2004; Pandiani et al., 1994) and likelihood of moving on to more independent living (Killaspy et al., 2013; Killaspy et al., 2019). Other personal and environmental factors can affect an individual’s experience of personal empowerment including their illness experience, restrictions imposed by compulsory treatment orders and rules within SA which can restrict choices and involvement in decision making (Brolin et al., 2018; Fossey et al., 2006; Nelson et al., 2001; Sandhu et al., 2017; Valdes-Stauber & Kilian, 2015). These environmental factors mean that staff have to uphold rules while also supporting individuals, limiting flexibility of approach which can hinder effective support for recovery (Bengtsson-Tops et al., 2014; Coffey et al., 2019; Nelson et al., 2007).

There is no indication in the included studies if participation in daily living, social participation and personal empowerment were assessed prior to people living in SA to inform decisions about which type of SA an individual moved to. Previous studies have shown that healthcare professionals can overestimate the level of support people with SMI require, which often differs from what the individual identifies as needing (Afilalo et al., 2015; Lasalvia et al., 2012; Piat et al., 2015). There is limited reporting on how an individual’s level of participation is considered when selecting SA, resulting in people with differing participation needs residing in the same types of SA. This can create a disparity between individuals’ needs, type of support provided and the extent to which people’s participation is enabled (de Heer-Wunderink et al., 2012; Sanches et al., 2019).

## Limitations

The number of studies included in the review are small and highlight that there is limited published research available focusing on formal support on participation for people with SMI living in SA. Due to the lack of appropriate data, the authors were unable to conduct a meta-analysis by estimating overall effect sizes. Instead, unstandardised effect sizes such as raw mean difference were used to explore if there was an association between level of support and participation for people with SMI, affecting the robustness of the results. The original aim of the review was to compare the impact of informal and formal care on people with SMI living in SA. The role of informal carers is under explored in current literature, even though informal care networks such as family involvement (Allen et al., 2013; Dorer et al., 2009; Fossey et al., 2006) or supportive neighbourhoods (Kriegel et al., 2019) are indicated as beneficial to people with SMI’s social participation and recovery. The review focuses on level of support and the authors acknowledge there are other factors that can influence participation for people with SMI living in SA including whether people are living in congregate settings or alone, and length of stay in accommodation which needs to be a consideration for future reviews.

## Conclusion

This review identified an association between participation factors and level of formal support for people with SMI, between accommodation with moderate support and accommodation with high support. People living in accommodation with medium support participated in more community occupations, a higher number of daily living activities and experienced greater personal empowerment. The results suggest that further exploration of how formal and informal support enables participation for people with SMI in SA to support their recovery is needed.

## Supplemental Material

sj-pdf-1-isp-10.1177_0020764020988576 – Supplemental material for The level of formal support received by people with severe mental illness living in supported accommodation and participation: A systematic review Click here for additional data file. Supplemental material, sj-pdf-1-isp-10.1177_0020764020988576 for The level of formal support received by people with severe mental illness living in supported accommodation and participation: A systematic review by Akkara Lionel Jose, Michele Harrison, Anusua Singh Roy, Linda Irvine- Fitzpatrick and Kirsty Forsyth in International Journal of Social Psychiatry
