# Supplementary material for: The level of formal support received by people with severe mental illness living in supported accommodation and participation: A systematic review
Source: Int J Soc Psychiatry. 2021 Jan 25;67(7):854–66. doi: 10.1177/0020764020988576 (PMC8559179; doi:10.1177/0020764020988576)
Supplement: sj-pdf-1-isp-10.1177_0020764020988576 – Supplemental material for The level of formal support received by people with severe mental illness living in supported accommodation and participation: A systematic review [file sj-pdf-1-isp-10.1177_0020764020988576.pdf]

Supplementary Material 1: Outcomes reported in included studies matched to participation factors

**Participation factor**	**Outcome reported in study**	**Measure used in study**
Social participation	Instrumental Roles (Nelson et al. 1997)	Total number of roles involved in
	Social contact (Fossey et al. 2006)	Life Skills Profile[1]
	Participation in community occupations (Dorer et al. 2009)	Survey developed by study authors
	Social Activity Factor (Shu et al. 2001)	Quality of Life Scale[2]
	Percentage of participants involved in daily activities (employed or student, working or enrolled in studies, attending a day centre, organised leisure/hobbies at least once a week, work training, cultural occupations; Eklund and Tjörstrand 2019)	Satisfaction with Daily Living Occupations[3]
Daily Living Functioning	Self-sufficiency (Kruzich and Berg 1985)	Self-sufficiency index developed by authors for study
	Independent Functioning (Nelson et al. 1987)	Independent Functioning Scale (adapted by authors from Rappaport et al. 1985)
	Self-care (Fossey et al. 2006)	Life Skill Profile[1]
	Percentage of participants involved in daily activities (doing household work, gardening or repairs, managing own personal hygiene on a daily basis, physical exercise; Eklund and Tjörstrand 2019)	Satisfaction with Daily Living Occupations[3]
Personal Empowerment	Mastery (Nelson et al, 1985)	Mastery Scale[4]
	Responsibility (Fossey et al. 2006)	Life Skills Profile[1]
	Autonomy (Shu et al. 2001)	Quality of Life Scale[2]

1. Rosen A, Hadzi-Pavlovic D, Parker G (1989) The Life Skills Profile: a measure assessing function and disability in schizophrenia. *Schizophrenia Bulletin, 15(2),* 325-37.
2. Yu, W.Y. (1995). The development of quality of life for mental illness patient. *Public Health in Taiwan, 22,* 29-39.
3. Eklund, M., Bäckström, M., & Eakman, A. (2014).Psychometric properties and factor structure of the 13-item satisfaction with daily occupations scale when used with people with mental health problems. *Health and Quality of Life Outcomes, 12(1),* 1–9.
4. Pearlin, L. I., & Schooler, C. (1978). The structure of coping. *Journal of Health and Social Behavior, 19*, 2–21.

Supplementary material 2: Search strategy

Database: Ebscohost - PsychINFO, PsychARTICLES, Medline, CINAHL ***Limited by Age over 18+,publications in English***	
**S29**	S11 AND S21 AND S22 AND S28
**S28**	S25 OR S27
**S27**	S23 OR S26
**S26**	occ* Participation OR process* skill* OR motor skill* OR cognitive ability OR roles OR occ* roles OR build* relation* OR organisation* skill* OR problem solving OR communicat* ability OR communicat* skill* OR Increase* responsibility OR improve* responsibility OR motivation to participate OR motivation to engage OR change in motivation
**S25**	S15 AND S24
**S24**	participation OR engag* OR involv*
**S23**	improved participation OR factors of participation OR participation OR level of participation OR empower* OR enabling relation* OR autonomy OR independ* OR promot* OR skill* OR abilit* OR occ* performance
**S22**	informal care* OR family OR relative* OR friend* OR support network
**S21**	S13 OR S14 OR S15 OR S17 OR S18 OR S19 OR S20
**S20**	facilitation OR encour* OR enabl*
**S19**	support OR care
**S18**	rapport OR rapport buil* OR buil* rapport
**S17**	S15 AND S16
**S16**	engage* OR interaction* OR environ*
**S15**	social
**S14**	S12 AND S13
**S13**	interaction OR relation* OR engage*
**S12**	staff OR care* OR professional*
**S11**	S9 AND S10
**S10**	S5 OR S7
**S9**	S1 AND S2
**S8**	S1 AND S2 AND S5 AND S7
**S7**	S4 AND S6
**S6**	shared
**S5**	S3 AND S4
**S4**	hous* OR accom* OR environment*
**S3**	support*
**S2**	client* OR resident* OR patient* OR service user*
**S1**	psyc* OR mental illness* OR mental health difficult* OR mental health

Database: ASSIA

(((noft(hous* OR accom* OR environment*) AND noft(shared OR support*)) AND (noft(psyc* OR mental illness* OR mental health difficult* OR mental health) AND noft(client* OR resident* OR patient* OR service user*))) AND ((noft(interaction OR relation* OR engage*)) OR (noft(staff OR care* OR professional*) AND noft(interaction OR relation* OR engage*)) OR (noft(social)) OR (noft(social) AND noft(engage* OR interaction* OR environ*)) OR noft(rapport OR rapport buil* OR buil* rapport) OR noft(support OR care) OR noft(facilitation OR encour* OR enabl*)) AND (noft(informal care*) OR noft(family) OR noft(relative*) OR noft(friend*) OR noft(support network)) AND ((noft(social) AND noft(participation OR engag* OR involv*)) OR ((noft (improved participation OR factors of participation OR participation OR level of participation OR empower* OR enabling relation* OR autonomy OR independ* OR promot* OR skill* OR abilit* OR occ* performance)) OR (noft(occ* Participation OR process* skill* OR motor skill* OR cognitive ability OR roles OR occ* roles OR build* relation* OR organisation* skill* OR problem solving OR communicat* ability OR communicat* skill* OR Increase* responsibility OR improve* responsibility OR motivation to participate OR motivation to engage OR change in motivation))))).

Supplementary material 3: Quality assessment of included studies

RTI item time bank

Study	SD	1	2	3	5	6	7	8	9	11	12	13	Interpret
Fossey et al. 2006	CS	N(low)	N(low)	N(low)	N/A	Y(low)	-	-	N(low)	Y(low)	Y(low)	N(low)	Low
Kruzich and Berg, 1985	C	N(low)	CD (unclear)	N(low)	N/A	N(high)	N(low)	CD (unclear)	N(low)	Y(low)	Y(low)	N(low)	Unclear
Dorer, Harries and Marston, 2009	CS	N(low)	N(low)	N(low)	N/A	N(high)	-	-	N(low)	Y(low)	Y(low)	N(low)	High
Eklund and Tjornstrand, 2019	CS	N(low)	Y(high)	N(low)	N/A	Y(low)	-	-	N(low)	Y(low)	Y(low)	N(low)	Low

**Question No.**	**Question**	**Type of bias idenitified**
1	Do the inclusion/exclusion criteria vary across the comparison groups of the study?	Selection bias
2	Does the strategy for recruiting participants into the study differ across groups?	Selection bias, confounding
3	Is the selection of the comparison group inappropriate?	Selection bias, confounding
5	Was the assessor not blinded to the outcome, exposure, or intervention status of the participants?	Detection bias
6	Were valid and reliable measures implemented consistently across all study participants to assess inclusion/exclusion criteria, physical activity outcomes, and potential confounders?	Detection bias, confounding
7	Was the length of follow-up different across study groups?	Attrition bias
8	In cases of high loss to follow-up (or differential loss to follow-up), was the impact assessed (e.g., through sensitivity analysis or other adjustment method)?	Attrition bias, detection bias
9	Are any important primary outcomes missing from the results?	Reporting bias
11	Are results believable taking study limitations into consideration?	Overall assessment
12	Were there any attempts to balance the allocation between the groups or match groups?	Confounding
13	Were important confounding variables not taken into account in the design and/or analysis?	Confounding

SD= Study design C= Cohort study CS= Cross-section study Y= Yes N=No CD= Cannot determine

ROBINS-I								
	**D1**	**D2**	**D3**	**D4**	**D5**	**D6**	**D7**	**Overall Risk of bias**
Nelson et al. 1985	Moderate	Low	Low	Low	Low	Low	Low	Moderate
Shu et al. 2001	Low	Low	Low	Low	Low	Low	Moderate	Moderate
Domains								
D1	Bias due to confounding							
D2	Bias due to selection of participants							
D3	Bias in classification of interventions							
D4	Bias due to deviations from intended interventions							
D5	Bias due to missing data							
D6	Bias due to measurement of outcomes							
D7	Bias in the selections of the reported result							
Low = low risk of bias; Moderate = moderate risk of bias								
